# Where Are the fMRI Correlates of Phosphene Perception?

**DOI:** 10.3389/fnins.2018.00883

**Published:** 2018-12-11

**Authors:** Tom A. de Graaf, Job van den Hurk, Felix Duecker, Alexander T. Sack

**Affiliations:** ^1^Section Brain Stimulation and Cognition, Department of Cognitive Neuroscience, Faculty of Psychology and Neuroscience, Maastricht University, Maastricht, Netherlands; ^2^Maastricht Brain Imaging Centre, Maastricht, Netherlands; ^3^Scannexus, Maastricht, Netherlands

**Keywords:** phosphenes, TMS, fMRI, null result, simultaneous

## Abstract

Pulses of transcranial magnetic stimulation (TMS) over occipital cortex can induce transient visual percepts called phosphenes. Phosphenes are an interesting stimulus for the study of the human visual system, constituting conscious percepts without visual inputs, elicited by neural activation beyond retinal and subcortical processing stages in the visual hierarchy. The same TMS pulses, applied at threshold intensity phosphene threshold (PT), will prompt phosphene reports on half of all trials (“P-yes”) but not on the other half (“P-no”). Contrasting brain activity (P-yes > P-no) can provide unique information on neural mechanisms underlying conscious percepts, as has been demonstrated by published EEG studies. Yet to our knowledge no articles reporting analogous contrasts with functional magnetic resonance imaging (fMRI) have been published. Since it seems unlikely that such studies have never been performed, this straightforward and technically feasible idea may have been explored in multiple failed, and unpublished, attempts. Here, we argue why such unsuccessful attempts, even small-scale, best be shared. We also report our own failed attempt to find phosphene-related activity in fMRI. Threshold phosphenes are weak percepts, and their detection subjective and difficult. If fMRI correlates of phosphenes are obtainable with this contrast, small-scale (‘pilot’) measurements may not be sufficiently powerful to detect them. At the same time, due to the challenges and costs involved in TMS-fMRI, attempts might not often get beyond the piloting stage. We propose that the only way out of this quandary is the communication and sharing of such unsuccessful attempts and associated data.

## Introduction

Transcranial magnetic stimulation (TMS) pulses applied to occipital cortex can elicit transient conscious visual percepts, known as phosphenes, without any visual stimulation. Participants can keep their eyes closed, be blindfolded (de Graaf et al., 2017a), or fixate with eyes open. The experience is fleeting, but spatially specific, retinotopically organized relative to the stimulated cortical area, and relatively reliable as observers gain experience reporting on their perception (Marg and Rudiak, 1994; Kammer, 1999; Kammer et al., 2005). The TMS intensity required to elicit phosphenes on half the trials is called the phosphene threshold (PT). Stimulating repeatedly at threshold opens up a range of interesting research questions, if brain activity can be measured concurrently. After all, a conscious visual percept will be induced directly at the level of visual cortex, on some trials, but not others, under identical conditions.

Contrasting brain activity in trials with reported phosphenes (P-yes) vs. trials without (P-no) could provide unique information on the neural basis of conscious vision. The search for the neural basis of consciousness continues to motivate development of new approaches and paradigms (Kim and Blake, 2005; de Graaf et al., 2017b). One problem in this search is that brain mechanisms related to conscious experience usually coincide with brain mechanisms related to unconscious processing (de Graaf et al., 2012; de Graaf and Sack, 2014; Gallotto et al., 2017). Ideally, one would bypass early stages of visual processing (retina, subcortical nuclei), or even induce conscious visual experiences without presenting visual inputs at all. These goals are approximated by the use of TMS-induced phosphenes as stimuli. Simultaneous neuroimaging might thus allow a ‘cleaner’ mapping of specifically awareness-related neural mechanisms as compared to alternative paradigms.

This potential is clearly demonstrated by studies with electroencephalography (EEG). Both neural responses to TMS pulses inducing phosphene perception, and neural mechanisms determining phosphene perception (i.e., activity prior to the pulses), have been reported. For instance, power in the alpha range (7–13 Hz) of the EEG spectrum predicts the perception of phosphenes (“P-yes” vs. “P-no” reports) both within (Romei et al., 2008a) and across (Romei et al., 2008b) participants. Taylor et al. (2010) directly compared the EEG response to a TMS pulse on P-yes and P-no trials. The neural signature of phosphene perception became apparent after 160 ms in widespread occipitoparietal areas. The authors concluded that widespread recurrent interactions are involved in phosphene perception, rather than a brief, early, local neural signature. In principle, this might encourage us to think that neural correlates of phosphene perception should be detectable with functional magnetic resonance imaging (fMRI) as well. A whole-brain approach could be relevant, since occipital and parietal phosphenes seem to correlate with differential activity in temporal and parietal cortices (Bagattini et al., 2015).

FMRI has better spatial resolution, as well as whole-brain coverage, and thus seems ideal to employ in the search for neural correlates of phosphene perception. Compared to other simultaneous TMS-fMRI studies, a phosphene experiment is relatively straightforward; no sensory stimuli are required, participants can simply lie on top of the MRI compatible coil, pressing buttons to report P-yes or P-no. The idea is straightforward and technically feasible, and it seems very likely that different labs over the last two decades have attempted to find fMRI correlates of phosphene perception. However, our search of the literature yielded one conference proceeding (Brodbeck et al., 2007), which does mention occipital activations related to phosphenes but does not seem to have resulted in a subsequent article publication (though see Halko et al., 2013, for discussion of results from one participant), and one published experiment (Caparelli et al., 2010) contrasting fMRI activations in phosphene perceivers vs. non-perceivers, not phosphene perception itself (P-yes vs. P-no trials). Caparelli et al. (2010) reported that occipital TMS pulses induced BOLD responses in a visual network whether or not participants could see phosphenes. A notable difference between phosphene perceivers and non-perceivers was increased activation at the stimulation site, similar to what has been shown for supra- vs. subthreshold TMS in the motor system (Bestmann et al., 2003). This cannot reveal neural correlates of phosphene perception directly, but it seems promising, as did the conference proceeding. So, after all this time: where are the fMRI correlates of phosphenes?

## The Hypothesis of a Carousel of Failures

If indeed labs capable of TMS-fMRI, over the years, have attempted P-yes vs. P-no TMS-fMRI experiments, then the lack of publications may suggest that these attempts did not fully succeed. One could ask, why were such failed attempts not published? TMS-fMRI is not trivial, for experimenter or participant, both when it comes to experimental setup, procedures, required expertise, and financial resources. So it is at least possible that previous attempts consisted of (possibly extensive) pilot measurements, which were subsequently abandoned due to lack of results. Null results are traditionally difficult to disseminate in any case, so null results from small-scale studies or even extensive pilot measurements might understandably not prompt experimenters to (attempt to) share their failed attempts with the community. This is likely a combination of a high likelihood of failure to be accepted for publication, a reluctance to draw conclusions from such small-sample null results, and limited impact even in case of publication.

But this scenario seems unfortunate. We suggest that failed attempts to find fMRI correlates of phosphenes should be disseminated, for three reasons. Firstly, a phosphene TMS-fMRI experiment requires quite some methodological development and expertise, and some intelligent experimental design. Details of the attempt might benefit future studies, either by offering clever solutions, or conversely by describing at least one setup/approach that did not seem to work and might thus be avoided. And even the simple knowledge that a lab has made such an attempt allows other interested parties to get in touch, communicate, or collaborate on new endeavors. Secondly, if data are linked to such dissemination, made accessible, then even small-scale studies could eventually lead to more meaningful larger-scale analysis approaches through data integration. Thirdly, the pragmatic argument (de Graaf and Sack, 2011, 2018): if no one shares their failed attempts at a straightforward project such as this, as a community we may keep going round and round in a carousel of uninformed failures. This seems a waste of time and resources.

Thus, we here report our own failed attempt to reveal fMRI correlates of TMS-induced phosphenes, in a small but relatively in-depth sample. We found essentially no results, even when using multiple, sophisticated, and liberal analysis approaches. Yet, our experimental design was sound and data quality as checked through temporal signal to noise ratio maps, as well as auditory responses to TMS pulses, seemed sufficient.

## A Failed Attempt to Find fMri Correlates of Phosphenes

In this section, we present an abbreviated report of our experiment, with more methodological and results details provided in Supplementary Material.

### Abbreviated Materials and Methods

We tested one experienced phosphene observer (author TdG) repeatedly, over 10 functional runs collected over three sessions, for an in-depth exploration of participant-level fMRI results. We tested three further experienced phosphene perceivers in single measurements of four functional runs, analyzed on the group-level.

Transcranial magnetic stimulation was applied in the scanner to occipital cortex, with participants lying on top of the coil in supine position. TMS intensity was informally calibrated individually and adapted across and within sessions to achieve and maintain the following conditions: TMS at PT, which should lead to approximately 50% P-yes trials and 50% P-no trials, TMS sub-threshold, and TMS supra-threshold. TMS intensity conditions were pseudo-randomized within runs, with computer control of intensity on a trial-by-trial basis. Per functional run, there were six trials for the sub- and supra-PT conditions, and 12 trials for the 100% PT condition. Thus, 24 trials in total for sub- and supra-PT conditions, and 48 for the 100% PT condition, per participant. In repeatedly scanned observer TG, we collected 60 trials in the sub- and supra-PT conditions and 120 trials in the 100% PT condition. We verified that in sub-, 100%-, and supra-PT conditions, the proportions of P-yes and P-no trials were approximately as intended (Supplementary Table S1).

We used flexible MRI coils to allow sufficient space for the TMS coil. Maps of temporal signal to noise ratios (tSNR) suggested acceptable data quality across the brain. Event-related average BOLD responses to TMS pulses showed clear responses in auditory cortex (Supplementary Figure S2). We performed analyses contrasting P-yes trials and P-no trials. Univariate and multivariate searchlight analyses (Kriegeskorte et al., 2006) were performed on the whole brain, and in anatomical atlas-based ROIs reflecting early visual areas (V1, V2, and V3). On the participant-level, we furthermore performed searchlight support vector machine mapping to differentiate P-yes and P-no trials, as well as ROI-based multi-voxel pattern analysis with the same goal. Univariate group-level analyses were performed at liberal thresholds in random-effects as well as fixed-effects models.

### Results

In none of the participant-level analyses, either in observer TdG or any other participant, did we find any significant voxel clusters surviving a liberal correction for multiple comparisons. Not in univariate analyses or multivariate analysis, not across the brain and not in ROI-based analysis.

On the group-level, one voxel cluster differentiated P-yes from P-no trials in one of the liberal analyses; fixed-effects whole brain general linear model, contrasting P-yes vs. P-no within the 100% PT condition. This cluster was in right frontal cortex and showed higher BOLD signal for P-no trials (Supplementary Figure S3). This cluster appeared to be largely in white matter, however, and is clearly not in visual processing streams most interesting for our research question.

### Discussion

The experimental setup and design of the measurements seemed largely successful, we collected as many trials as feasible (120 event-related trials in 100% PT condition of observer TdG alone), and performed liberal and exploratory analyses to potentially see effects on the single-participant or group level. Yet, at least within occipital cortex, but really across the whole brain, we found no convincing activations that might underlie phosphene perception.

## Final Remarks

Why might it be difficult to find neural mechanisms differentiating P-yes from P-no trials? Caparelli et al. (2010) reported that occipital TMS pulses induce stronger local BOLD responses in phosphene perceivers, and a reasonable hypothesis might be that P-yes trials in contrast to P-no trials should reveal the same. One possibility is that the local BOLD response reported by Caparelli et al. (2010) does not reflect phosphene perception, but some intrinsic ability to see phosphenes. Perhaps fMRI simply cannot reveal the neural processes that differentiate P-yes from P-no. Neural events might be quick and short-lasting (Mazzi et al., 2017), and fMRI has low temporal resolution. More generally, the BOLD response does not capture all aspects of brain function. An alternative possibility is that fMRI *can* in principle reveal phosphene correlates, but these simply constitute a very small/weak effect. Phosphene perceivers as opposed to non-perceivers may have very different and salient perceptual experiences. Yet, when it comes to P-yes vs. P-no, the judgment is difficult and subjective. From experience, in some trials there clearly is a phosphene, or no phosphene at all. But in many trials, the decision is not so easy; stimulation at threshold may elicit very weak and fleeting phosphene experiences, not always straightforward to judge as P-yes or P-no.

Perhaps it ultimately is a case of a weak effect requiring larger numbers of trials and participants to detect. A problem may then be that there are technical and financial challenges involved in TMS-fMRI, and many attempts may not proceed beyond a limited set of measurements (pilot) if results are not encouraging. That is even more reason to share experiences and attempts to perform experiments such as these. We invite the community to contribute their attempts, past or future, to make transparent what has been tried and in which labs. Knowledge could be exchanged, and perhaps data pooled to perform further analyses. Perhaps together, we can find the fMRI correlates of phosphenes.

## Ethics Statement

This study was carried out in accordance with the recommendations of the local Ethical Review Committee Psychology and Neuroscience with written informed consent from all subjects. All subjects gave written informed consent in accordance with the Declaration of Helsinki. The protocol was approved by the local Ethical Review Committee Psychology and Neuroscience.

## Author Contributions

All authors contributed to writing the manuscript. TG, FD, and AS designed the experiments. TG and FD performed the measurements. JvdH performed the analyses.

## Conflict of Interest Statement

The authors declare that the research was conducted in the absence of any commercial or financial relationships that could be construed as a potential conflict of interest.
