# Supplementary material for: Where Are the fMRI Correlates of Phosphene Perception?
Source: Front Neurosci. 2018 Dec 11;12:883. doi: 10.3389/fnins.2018.00883 (PMC6297746; doi:10.3389/fnins.2018.00883)
Supplement: Supplementary file 1 [file Table_1.DOCX]

**Supplementary Material**

**Where are the fMRI correlates of phosphene perception?**

*de Graaf, TA* 1,2*., van den Hurk, J. 3, Duecker, F. 1,2, Sack, AT. 1,2.

**Supplementary Materials and Methods**

*Participants*

One experienced phosphene perceiver (author TG) was measured across three different sessions to collect a sizeable dataset for in-depth within-participant analysis. Three additional volunteers with experience in phosphene perception were measured in single sessions. All were screened for fMRI and TMS safety. Participants were compensated for their time with monetary coupons. The experiment was approved by the local ethics committee.

*Procedure and design*

Phosphene threshold with the TMS equipment to be used inside the MR environment was informally determined before participants entered the scanner, and the phosphene hotspot relative to skull landmarks noted for quick repositioning in supine position on the scanner bed. For stimulation during scanning, the TMS coil was placed inside the head coil, so that participants could lie on top of it in a comfortable supine position.  Once participants were lying comfortably, the coil was moved slightly, and/or they made small adjustments with their heads, to relocate the phosphene hotspot as pulses were administered. Phosphene threshold was redetermined while stimulating at the final and fixed occipital target site.

Based on this phosphene threshold (PT), the three TMS intensities to be used in the experimental session were determined: sub-, 100%, or supra-PT. Due to the exploratory nature of this study, though in principle sub- and supra-PT conditions involved TMS intensities of 80% of PT and 120% of PT, in a few runs we used 90% PT and 110% of PT. This appears of little consequence since we focused analyses on direct contrasts of P-yes and P-no trials, and particularly within the 100% PT condition, so we continue to refer to sub- and supra-PT conditions. We checked behaviorally whether reports of phosphenes were less common in the sub-PT condition, occurred approximately half the time in the 100% PT condition, and were most common in the supra-PT condition. See Table S1 for percentages of reported phosphenes for all observers across all conditions. Since the most interesting contrast afforded by this design is the comparison of BOLD activity in P-yes and P-no trials, within the 100% PT condition, because only conscious percept and/or response criteria could be different between these trials, we always included twice as many 100% PT trials as compared to the sub- and supra-PT conditions.

|  | TMS intensity | |  |
| --- | --- | --- | --- |
| participants | sub-PT | 100% PT | supra-PT |
| 1 | 25% | 52% | 83% |
| 2 | 38% | 52% | 88% |
| 3 | 38% | 73% | 88% |
| 4 | 5% | 61% | 92% |
| Table S1: percentages of trials with reported | | | |
| phosphenes per participant and TMS intensity. | | | |
| Subject 4 is author 'TG' with 10 functional runs. | | | |

Per experimental run, there were 6 trials for the sub- and supra-PT conditions, and 12 trials for the 100% PT condition. Per participant, there were four functional runs, i.e. 24 trials in total for sub- and supra-PT conditions, and 48 for the 100% PT condition. Observer TG was measured in three sessions for a total of 10 functional runs, leading to 60 trials in the sub- and supra-PT conditions and 120 trials in the 100% PT condition.

Within a functional run, the order of trials from conditions was pseudorandomized. This means the intensity for TMS pulses was determined on a trial-by-trial basis, controlled through serial communication with the TMS device by a controlling stimulation PC. The inter-pulse interval was 16.5 seconds on average, jittered by 1.5 seconds. After each TMS pulse, participants indicated by means of button presses whether they perceived a phosphene on that trial or not. Thus, P-yes and P-no conditions were labeled post-hoc based on their responses.

*(f)MRI parameters*

Anatomical and functional scans were acquired using a 3T MAGNETOM Prisma Fit MRI scanner (Siemens, Erlangen, Germany). To afford enough physical space inside the head coil for both participant heads, cushioning materials, and the TMS coil, we used two Siemens Flex 4 head coils and a CIVCO head coil mount. These allow quite some flexibility for comfortable and spacious positioning, while retaining acceptable temporal signal-to-noise ratios (tSNR, see Supplementary Results).

Each functional run consisted of 274 T2*-weighted echoplanar images (EPIs) (multiband factor 2, resolution 3x3x3 mm, 44 slices, interslice gap 0 mm, TR = 1500 msec, TE = 30 msec, 72x72 matrix). In addition to the functional images, we collected a high-resolution T1-weighted anatomical scan for each participant (256 slices, resolution 1x1x1 mm, TR = 2250 msec, TE = 2.21 msec, 256x256 acquisition matrix). Within the TR, the slice acquisition took 1400 ms, leaving a 100ms acquisition gap. TMS pulses were administered at 1450ms, thus 50 ms prior to the onset of the next volume. Pilot testing confirmed that this interval was sufficient to avoid artifacts in subsequent EPI measurements.

*TMS parameters*

TMS pulses were administered by a Magventure X100 device (MagVenture, Denmark), through an MRI-compatible coil (MRi-B90 II, MagVenture, Denmark). The orientation of the figure-8 coil was such that the stimulating currents were in the lateral-medial plane, in other words with the imaginary handle (absent in an MRI-compatible coil) at a 90-degree angle to the spine. TMS intensity was individually calibrated based on PTs (see Procedure above). If participants moved between runs and PT was changed, or the responses indicated that 100% PT trials yielded a disproportionate number of P-yes or P-no responses, TMS intensities for the different conditions could be adapted between runs. Average phosphene thresholds in terms of percentage maximal machine output for the three single-session participants were: 42%, 58.5%, 60%, for observer TG it was 85.4%.

*Analyses*

The goal of this pilot study was to evaluate whether we could find any indication of BOLD modulations in occipital cortex, or elsewhere, by the presence versus absence of a phosphene experience. Of course, to establish such modulations, rigorous analysis and appropriate statistics are required. However, since we ultimately report here a failure to find any such modulations in this small-scale pilot study, the opposite seems important: we explored the data with statistical thresholds generally considered overly liberal, and report that *still* we find no evidence for BOLD modulations in occipital cortex.

Using BrainVoyager 20.6 (Brain Innovation, Maastricht, The Netherlands) we performed univariate analyses through a general linear model, both in a random-effects model (conventional, to reveal effects on the population level) and in a more liberal fixed-effects model taking each acquired run as equivalent data points, thus all 3 x 4 + 1 x 10 runs together to possibly reveal effects on the sample level. We also contrasted P-yes and P-no trials in ROIs based on a functional atlas (Rosenke et al., under review) capturing early visual areas V1, V2, and V3. Whole-brain results were liberally corrected for multiple comparisons using a voxel-level threshold of P=0.001 and Monte Carlo estimated voxel cluster thresholds (Forman et al., 1995).

Using custom scripts developed and run in Matlab 2016a (Mathworks Inc, Natick, MA) utilizing functionality of the Neuroelf toolbox (www.neuroelf.net) we furthermore performed multivariate analyses on the participant level. First, we employed a so-called searchlight approach (Kriegeskorte et al., 2006). This descriptive method uses a spherical ‘searchlight’ that centers on every voxel of the cortical volume and gives an estimate of the presence of information in the spherical surround. On a single subject level, the algorithm targets all voxels individually and selects the voxels within the searchlight sphere with predefined radius surrounding the centered voxel. The voxels within this sphere are submitted to a binary classification algorithm that performs classification on a dataset by placing all cases in a multidimensional space. Each individual case (or example) is expressed as a vector of N features in the N-dimensional space. The algorithm then defines an optimal separation boundary, or hyperplane, between the two classes, given the training data. The generalizability of the trained classifier is subsequently assessed by feeding the independent and unlabeled test trials to the algorithm. The accuracy at which the classifier is able to determine the correct labels from these trials given only the response patterns is an indication of successful learning of the algorithm. This, in turn, reflects a meaningful difference in spatial patterns of neural activity elicited by the two conditions. The resulting prediction accuracy is returned to the center voxel, and this procedure is repeated for all voxels in a volume.

In this experiment, the analysis was performed using custom written MATLAB. First, for each voxel, individual responses to the experimental trials were estimated by fitting a double-gamma hemodynamic response function (HRF) to the voxel’s time course, using the resulting beta as trial estimate. Then, at each voxel, a sphere was defined with a radius of 3 voxels surrounding the center voxel. For each sphere, this resulted in a matrix with dimensions VxT, where V represents the number of voxels within the sphere, and T stands for the number of trials. The trials were labeled according to their corresponding condition (P-yes, P-no) and submitted to a linear support vector machine classifier (Mourão-Miranda, Bokde, Born, Hampel, & Stetter, 2005), using a leave-1-run-out cross-validation. In addition, 100 permutations were run per spherical voxel cluster to rank the true predication accuracy against. The map of statistically significant (P<0.05) voxels was inspected both with and without false discovery rate correction for multiple comparisons. Aside from this exploratory multivariate approach, we also directly performed lSVM analysis on anatomically defined ROIs, for V1, V2, V3, in left and right hemispheres separately, using the same parameters as for the searchlight.

**Supplementary Results**

*Data quality*

Figure S1 below shows tSNR maps for all four observers (repeatedly scanned participant TG is top-right). The flexible MR coils resulted in lower tSNR than is obtained with conventional specialized MR headcoils, but not to an extent that it should explain a complete lack of results. The tSNR seems acceptably evenly distributed across the brain based on these slices.


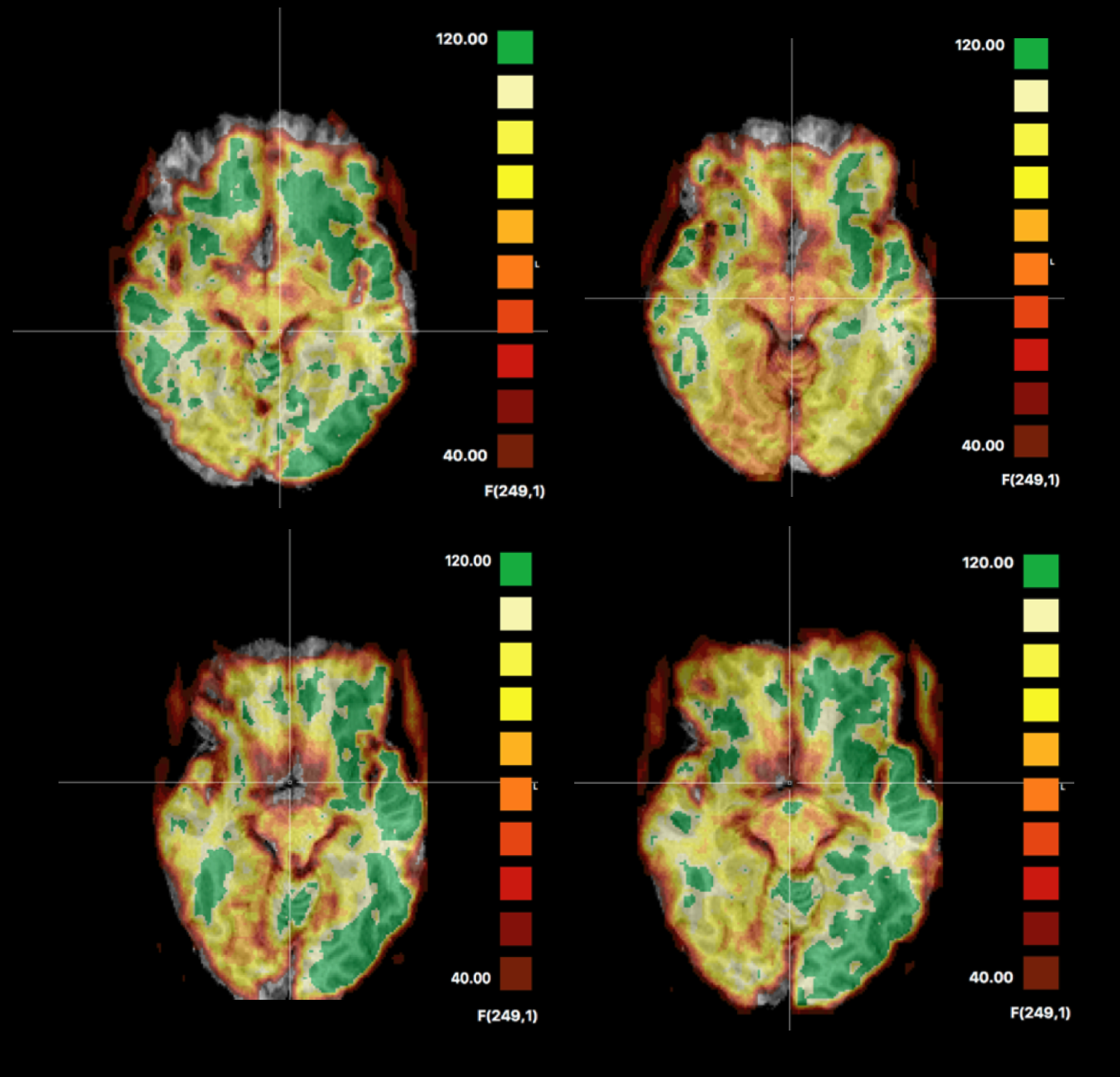


*Figure S1. tSNR maps in cross-sections for all four participants.*

We also ensured that our lack of results could not be attributed to errors in functional-anatomical coregistration or stimulation protocols, by evaluating the responses in auditory cortex to TMS pulses. The mechanical vibrations associated with magnetic pulses create loud ‘clicks’, which should induce detectable event-related BOLD responses in auditory cortices despite auditory noise of EPI acquisitions. In a template brain, we manually created a bilateral ‘auditory cortex’ region of interest (ROI), liberally marking voxels on and around Heschl’s gyrus. For those voxels, a fixed-effects ROI GLM showed a clear response to TMS pulses (full model vs. baseline; p < 0.0001). Separately for the three TMS intensity conditions, collapsed over P-yes and P-no trials, Figure S2 displays the fixed-effects group-level event-related average (i.e. BOLD response to TMS pulses). For each TMS intensity, auditory responses are clear, with the expected peak after approximately 4.5-6 seconds.


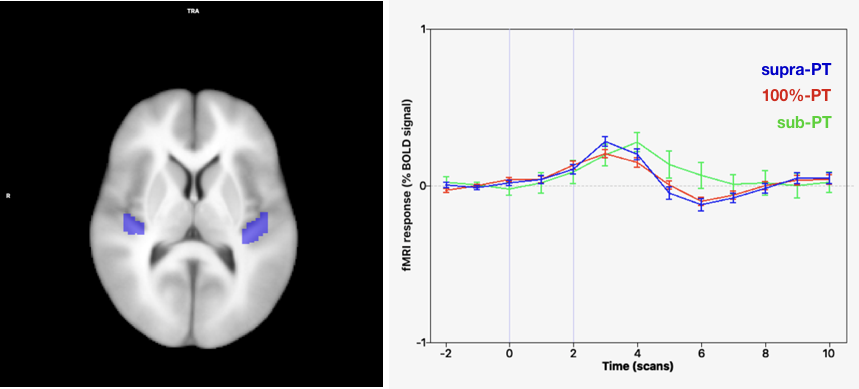


*Figure S2. Auditory responses to TMS pulses*

*Shown on the left is a cross-section of manually demarcated auditory cortices on a template brain, shown on the right are the event-related average responses to TMS pulses for the three TMS intensity conditions across all runs of all participants.*

*Individual Observers*

We stimulated one experienced phosphene perceiver with single TMS pulses across ten fMRI runs in three different sessions. In univariate analyses we compared across the brain whether any voxels were differentially activated by TMS pulses followed by a phosphene report (P-yes) as opposed to a report that no phosphene was perceived (P-no), collapsed across all three TMS intensity conditions. We observed no meaningful activations anywhere in the brain, and indeed no voxels survived a cluster-level threshold estimation procedure of correcting for multiple comparisons. Also when contrasting specifically P-yes trials to P-no trials within the 100% phosphene threshold condition (PT), no activation was observed in occipital cortex or elsewhere.

Whole-brain multivariate analysis was done in all individual participants, with a searchlight support vector machine trained to differentiate P-yes from P-no trials in the 100% PT condition. In no participant, including the extensively scanned experienced observer, did we observe any voxels that could differentiate these trials better than chance. We moreover performed a region-of-interest based multivariate analysis to differentiate P-yes from P-no trials using all voxels in anatomical atlas-based V1, V2, V3. In no subjects, for any of these ROIs, did we see any statistically significant result.

*Group analyses*

We performed the same contrasts to the sample as a whole, including the participant scanned repeatedly, in both a fixed-effects and random-effects general linear model analysis. No occipital activations were observed in either model, not for the contrast of all P-yes vs P-no trials, nor for the same contrast within only the PT condition. Outside occipital cortex, only in the fixed-effects analysis contrasting P-yes to P-no trials of the 100% PT condition did we see one activated cluster (11 functional voxels), see Figure S3. This was in right frontal cortex (TAL X = 25, Y = 42, Z = 4) and did survive (our very lenient) multiple comparisons correction. This cluster was more active for P-no trials than P-yes trials, suggesting to us that, if it is meaningful at all, it may be related to response criteria or decisions, not likely the conscious visual experience of a phosphene.


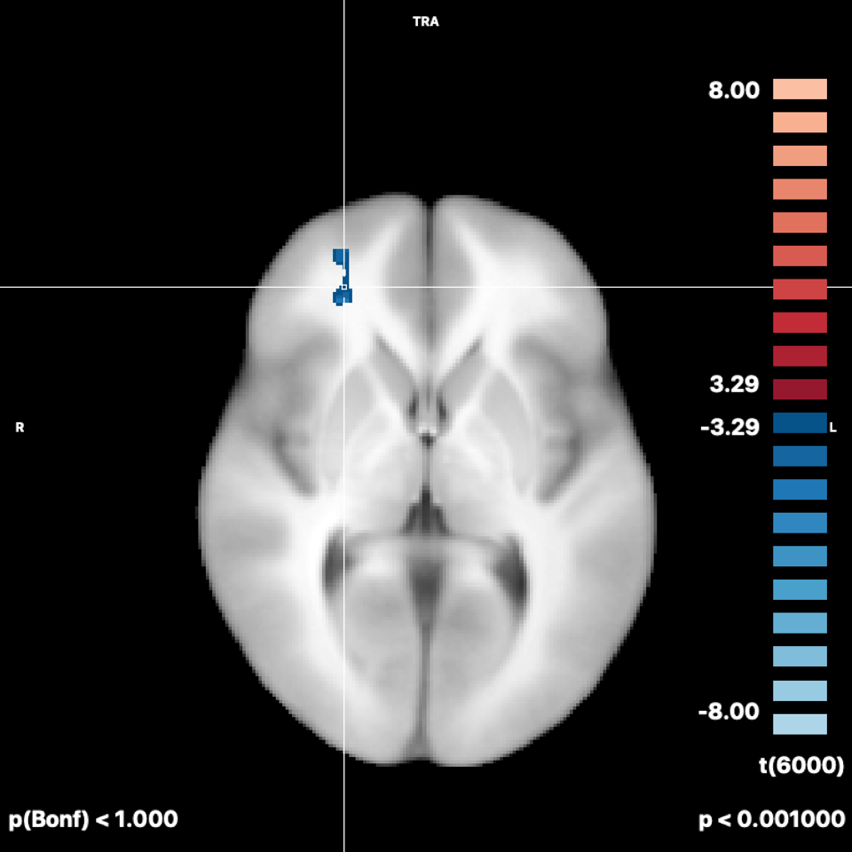


*Figure S3. Frontal cluster P-no > P-yes*

*The crosshairs indicate the position for which Talairach coordinates are reported in supplementary text. The activation map is superimposed on a template brain. Given the very liberal analysis yielding this cluster, and its apparent location in white matter, we caution against over interpretation.*

**Supplementary references**

Forman SD, Cohen JD, Fitzgerald M, Eddy WF, Mintun MA, Noll DC. Improved assessment of significant activation in functional magnetic resonance imaging (fMRI): use of a cluster-size threshold. Magn Reson Med. 1995;33:636–647

Kriegeskorte, N., goebel, R., & Bandettini, P. (2006). Information-based functional brain mapping. *Proceedings of the National Academy of Sciences of the United States of America*, *103*(10), 3863–3868. <http://doi.org/10.1073/pnas.0600244103>

Mourão-Miranda, J., Bokde, A. L. W., Born, C., Hampel, H., & Stetter, M. (2005). Classifying brain states and determining the discriminating activation patterns: Support Vector Machine on functional MRI data. *NeuroImage*, *28*(4), 980–995. http://doi.org/10.1016/j.neuroimage.2005.06.070
